# Work ability and quality of working life in atopic dermatitis patients treated with dupilumab

**DOI:** 10.1111/1346-8138.15939

**Published:** 2021-05-19

**Authors:** Angela L. Bosma, Wouter Ouwerkerk, Merve Günal, Ariënna M. Hyseni, Bernd W. M. Arents, Louise A. A. Gerbens, Maritza A. Middelkamp‐Hup, Angela G. E. M. de Boer, Phyllis I. Spuls

**Affiliations:** ^1^ Department of Dermatology Amsterdam Public Health Research Institute Amsterdam Institute for Infection and Immunity University of Amsterdam Amsterdam UMC, Location Academic Medical Center Amsterdam The Netherlands; ^2^ National Heart Centre Singapore Singapore; ^3^ Dutch Association for People with Atopic Dermatitis Nijkerk The Netherlands; ^4^ Coronel Institute of Occupational Health Amsterdam Public Health Research Institute University of Amsterdam Amsterdam UMC, Location Academic Medical Center Amsterdam The Netherlands

**Keywords:** atopic dermatitis, dupilumab, occupation, routine clinical care, work

## Abstract

Atopic dermatitis is associated with work productivity loss. Little is known about how patients perceive their work ability and quality of working life, and how this is affected by treatment. Our primary objective was to investigate work ability and quality of working life at baseline and during treatment in the long term. A registry‐embedded prospective observational cohort study was conducted consisting of patients with atopic dermatitis starting dupilumab in routine clinical care. The instruments used were the Work Ability Index (WAI; questions 1, 2, and 3) and the Quality of Working Life Questionnaire (QWLQ). Ninety‐three patients were included of whom 72 were (self‐)employed (77%). From baseline to 48 weeks, the mean WAI‐1 score (general work ability, range 0–10) improved from 6.8 (±2.0) to 7.9 (±1.3), WAI‐2 (physical work ability, range 1–5) from 3.7 (±0.9) to 4.3 (±0.7), and WAI‐3 (mental/emotional work ability, range 1–5) from 3.4 (±0.9) to 3.9 (±0.8) (*p* = 0.001, *p* = 0.005, *p* < 0.001, respectively). The mean QWLQ total score improved from 74.0 (±9.1) to 77.5 (±9.6) and subscale “Problems due to health situation” improved from 37.4 (±22.3) to 61.5 (±23.1) (range 0–100; *p* = 0.032, *p* < 0.001, respectively). In conclusion, patients with moderate‐to‐severe atopic dermatitis starting dupilumab report decreased work ability and quality of working life, mainly due to health‐related problems. Significant improvement of work ability and quality of working life is observed with dupilumab treatment.

## 1 INTRODUCTION

Atopic dermatitis (AD) is a common chronic dermatological condition that is associated with impairment of quality of life and work productivity.^1^ Skin diseases were found the 18th leading cause of global disability‐adjusted life years. Excluding mortality, skin diseases were the fourth leading cause of disability worldwide.^2^ Of all skin diseases, AD has the highest non‐fatal health burden.^3^, ^4^ AD is associated with sick leave, change or loss of job, and receiving disability pensions.^5^ Data from the TREATgermany registry has shown that moderate‐to‐severe AD has a substantial adverse economic impact with a mean productivity loss of almost 10%.^6^ Patients with AD using systemic treatment are found to incur considerable direct costs as well as indirect costs resulting from productivity loss.^7^

Little is known about how AD patients perceive their work ability and quality of working life (QWL). The Work Ability Index (WAI) was developed to investigate how long people are able to work and to what extent they are able to work depending on work content and demands. The WAI is considered reliable and valid, and has become a common tool to investigate work ability in research worldwide.^8^, ^9^ QWL is defined by the experiences and perceptions in the work situation.^10^ The Quality of Working Life Questionnaire (QWLQ) was developed to assess subjective work outcomes in employed cancer patients.^10^, ^11^ In contrast to other questionnaires it was not developed for healthy employees or particular occupations.^12^ Adequate internal consistency, construct validity and reproducibility, as well as sufficient responsiveness and interpretability were found in cancer survivors.^12^, ^13^ To date, WAI or QWLQ have never been used in the AD population.

The aim of this study was to generate new knowledge on work‐related outcomes in AD, focusing on work ability and QWL in particular. The primary objective was to investigate the work ability and QWL of AD patients at baseline and during dupilumab treatment using WAI and QWLQ scores. The secondary objectives were to explore associations between change in QWLQ (from baseline to 48 weeks) and baseline characteristics, and to explore the convergent validity of the QWLQ.

## 2 METHODS

### 2.1 Study design and patient population

We conducted a registry‐embedded prospective observational study in patients with AD based on the UK Working Party criteria.^14^ Patients of the Department of Dermatology of Amsterdam UMC starting treatment with dupilumab in context of routine clinical care, indicating moderate‐to‐severe disease, were included from November 2017 to February 2020. Six patients refrained from participation and informed consent was obtained from all participants. Apart from the requirement for informed consent, there were no exclusion criteria. A subset of TREAT NL registry data was used.^15^ Patients starting treatment with other systemic immunomodulating therapies or phototherapies, which are also included in the TREAT NL registry, were not included in this study as the numbers were low. At baseline and every 24 weeks thereafter, outcome data was collected (see “Study outcomes”). The study was exempted from evaluation by our local medical research ethics committee (W18_097#18.123). The study was carried out in accordance with the provisions of the Declaration of Helsinki.

All patients met the national criteria for dupilumab as determined by the Dutch Society of Dermatology which stipulate a failed treatment episode (ineffectiveness or adverse events) with one or more conventional systemic therapy(ies) prior to starting dupilumab.^16^ In two patients, dupilumab was prescribed off‐label at the time, as they were 17 years old. All other patients were adults. Patients started with an initial loading dose of 600 mg, followed by 300 mg dupilumab injections every 2 weeks. In our analyses we included patients while receiving dupilumab, regardless of dosing interval deviations and follow‐up duration. In accordance with (daily practice), patients were allowed to continue using conventional systemic treatment in a tapering schedule and to use topical treatments (e.g., corticosteroids and calcineurin inhibitors).

### 2.2 Study outcomes

Data collection was based on the TREAT core dataset.^15^, ^17^, ^18^ The following baseline characteristics were retrieved: demographics (sex, age, ethnicity, educational status: ISCED [International Standard Classification of Education] classification), health‐related characteristics (disease duration, comorbidities, outpatient daycare treatments, and hospitalizations for AD) and work‐related characteristics (work status: Eurostat classification [e.g., [self‐/un]employed], number of days lost from usual activities [e.g., work, study], problems at work [e.g., fatigue], reasons for not working [e.g., retired]).

As part of this study, we implemented the WAI and QWLQ in the Amsterdam UMC dataset (Appendix S1 and S2).^8^, ^12^ The first three WAI questions were used (i.e., WAI‐1, WAI‐2, WAI‐3), giving insight into patient‐reported general, physical, and mental work ability, respectively. General work ability (WAI‐1) was assessed in comparison to best work ability ever, on a scale of 0 (worst) to 10 (best). Five‐point Likert scales were applied to assess work ability with respect to physical (WAI‐2) and mental/emotional demands of the work (WAI‐3). QWLQ is a 23‐item questionnaire focusing on five subscales: (i) Meaning of work; (ii) Perception of the work situation; (iii) Atmosphere in the working environment; (iv) Understanding and recognition in the organization; and (v) Problems due to the health situation, which are scored on a 6‐point Likert scale. Higher scores correspond with better QWL, ranging 0–100.^12^ These subscales are considered to capture the complete scope of QWL and were based on literature and focus group discussions.^12^ In cancer survivors, improvement of more than 3.9 of the QWLQ total score after an intervention is considered clinically meaningful.^13^ For the WAI, the clinically meaningful change in score is unknown.

Correlation was investigated between QWLQ and patient‐reported outcome measurements (PROMs) indicating symptoms and quality of life in AD,^18^, ^19^ that were also collected every 24 weeks: Numerical Rating Scale (NRS) peak pruritus past 24 h (0–10),^20^ NRS mean pruritus past 7 days (0–10),^21^ Visual Analog Scale (VAS) peak pain past 24 h (0–10), VAS mean sleep loss past 3 days (0–10), Patient Global Assessment (PGA: 0–4), Patient‐Oriented Eczema Measure (POEM: 0–28),^22^ Dermatology Life Quality Index (DLQI: 0–30),^23^ and EuroQol‐5 dimensions–5 level health score (EQ‐5D‐5L health score: 0–100).^24^ All were available in Dutch and English and administered at the same time.

When more than 15% of patients achieve the lowest or highest possible score on the QWLQ or its subscales, this is considered a floor or ceiling effect.^25^, ^26^

### 2.3 Statistical analyses

Patient characteristics and scores were summarized using descriptive statistics and paired *t*‐tests as appropriate. A linear mixed‐effects model, with patients as random effect, was used to model scores over time up to 96 weeks as latest time point.

To explore associations between baseline characteristics and change in QWLQ from baseline to 48 weeks, we first imputed missing values five times using multi‐chain Monte Carlo methods Gibbs sampling.^27^ Afterwards, we performed multivariable linear regression analysis with stepwise backwards selection using Akaike Information Criterion. The stepwise backward regression uses 1000 bootstrap samples to get a robust selection of important patient characteristics associated with change in QWLQ. We performed the regression analysis in all five imputed datasets and only selected patient characteristics if they were selected in all five analyses. Patients with missing data on QWLQ at baseline or 48 weeks were excluded in these analyses.

Convergent validity is assessed by means of hypothesis testing: determining whether scores of an instrument correlate with other instruments in a way that one would expect.^28^ Hypothesis testing is part of investigating construct validity, as proposed by the COSMIN taxonomy of measurement properties.^29^ Our hypothesis was that a correlation (*r*) > |0.40| exists for EQ‐5D‐5L health score, POEM, DLQI, PGA, NRS pruritus, VAS pain, and sleep loss, indicating moderate‐to‐strong correlations (|0.20|–|0.39|: weak; |0.40|–|0.59|: moderate; |0.60|–|0.79|: strong).^25^ Spearman correlations were used to assess the correlation between QWLQ total score, subscale “Problems due to the health situation” and these constructs.

Analyses were performed using SPSS 25.0 (IBM) and R version 4.0.2 (Foundation For Statistical Computing). In all analyses, results were considered statistically significant at *p* < 0.05.

## 3 RESULTS

This study included 93 patients with baseline characteristics shown in Table 1. The majority of patients was male (58%) and white (76%). The average age (±standard deviation) was 43 (±15) years. The mean disease duration was 39 (±15) years. The majority had allergic comorbidities (up to 68%). Educational status ranged from ISCED 1 (primary education) to ISCED 8 (doctoral level). There were 53 (57%) patients employed, eight (9%) self‐employed, seven (8%) retired, one (1%) student, two (2%) unemployed, four (4%) both employed and self‐employed, seven (8%) both employed and student, and 11 (12%) received a disability pension. Of the 72 working patients (either employed or self‐employed), 46 (64%) reported to experience problems at work, with a combination of problems (including pruritus, fatigue, pain, and psychological complaints) being most common. In total, 54 patients reported days lost from usual activities in the past 3 months (58%) with a median of 4 days/month (25th–75th percentile [interquartile range [IQR]], 1–7). The median days lost from usual activities was 3.5 (IQR, 1–5) in working and 16.3 (IQR, 2.5–30) in not working patients (*p* = 0.01).

At baseline, the median EASI was 14.6 (range, 1.2–60.3) and 29% of patients had severe disease according to Investigator Global Assessment (IGA). At 48 weeks, the median EASI improved to 5.4 (range, 0.1‐25.2) and none of the patients had severe disease according to IGA.

**TABLE 1:** Patient characteristics at baseline

Demographic, health‐, and work‐related characteristics	TREAT NL cohort (n = 93)
Demographic characteristics	
Sex, n (%)	
Male	54 (58)
Female	39 (42)
Age in years, mean ± SD	43 ± 15
Ethnicity, n (%)	
White (Europe, Russia, Middle East, North Africa, USA, Canada, Australia)	71 (76)
African‐other, Afro‐Caribbean	3 (3)
Afro‐American	0 (0)
Asian‐Chinese	4 (4)
South‐Asian (India, Pakistan, Sri Lanka, Nepal, Bhutan, Bangladesh)	6 (7)
Asian‐other (Korea, China north of Huai River)	5 (5)
Japanese	0 (0)
Hispanic or Latino	0 (0)
Mixed^a^	4 (4)
Other	0 (0)
Educational status: ISCED classification, n (%)	
ISCED 0: Early childhood education	0 (0)
ISCED 1: Primary education	3 (3)
ISCED 2: Lower secondary education	15 (16)
ISCED 3: Upper secondary education	22 (24)
ISCED 5: Short‐cycle tertiary education	16 (17)
ISCED 5: Short‐cycle tertiary education	4 (4)
ISCED 6: Bachelor’s or equivalent level	22 (24)
ISCED 7: Master’s or equivalent level	8 (9)
ISCED 8: Doctoral or equivalent level	3 (3)
Health‐related characteristics	
Disease duration in years, mean ±SD	39 ± 15
Allergic comorbidities, n (%)	
Asthma^b^	54 (58)
Allergic rhinoconjunctivitis^b^	56 (60)
Atopic eye disease^b^	12 (13)
Eosinophilic esophagitis^b^	0 (0)
Allergic contact dermatitis^1^ ^c^	63 (68)
Food allergy^2^ ^d^	53 (57)
Treatment at outpatient daycare treatment unit in the past year, n (%)	18 (19)
Treatment at outpatient daycare treatment unit in the past year in cumulative days, median (IQR)^3^	5.5 (3–13.5)
Hospitalization for atopic dermatitis in the past year, n (%)	7 (8)
Hospitalization for atopic dermatitis in the past year in cumulative days, median (IQR)	7.0 (2–14)
Work‐related characteristics	
Work status: Eurostat classification, n (%)	
Employed	53 (57)
Self‐employed	8 (9)
Disability pension (unable to work)	11 (12)
Retired	7 (8)
Student or pupil	1 (1)
Engaged on home duties	0 (0)
Unemployed	2 (2)
Employed and self‐employed	4 (4)
Employed and student or pupil	7 (8)
Working patients, n (%)^e^	72 (77)
Patients that reported problems at work, n (%)^4^ ^f^	46 (64)
Combination of problems^g^	15 (21)
Psychological problems	9 (13)
Pain	8 (11)
Fatigue	4 (6)
Pruritus	3 (4)
Receiving insufficient understanding from the working environment	2 (3)
Inconsistent course of illness	1 (1)
Other^h^	4 (6)
Patients that reported days lost from usual activities (e.g., work, study), n (%)^i^	54 (58)
Average number of days lost from usual activities per month, median (IQR)^5^ ^j^	4 (1–7)
Working patients that reported days lost from usual activities, n (%)^k^	43 (60)
Average number of days lost from usual activities per month in working patients, median (IQR)^6^	3.5 (1–5)
Not working patients that reported days lost from usual activities, n (%)^l^	11 (52)
Average number of days lost from usual activities per month in not working patients, median (IQR)^7^	16.3 (2.5–30)
Patients that reported reasons for not working, n (%)^l^	15 (71)
Retired	6 (29)
Incapacitated for work because of experienced limitations due to atopic dermatitis	3 (14)
Incapacitated for work because of other reasons	3 (14)
Incapacitated for work because of a combination of atopic dermatitis and other reasons	1 (5)
Unemployed	2 (10)

Note

Missing data: ^1^n = 1, ^2^n = 1, ^3^n = 2, ^4^n = 4, ^5^n = 2, ^6^n = 1, ^7^n = 1.

Abbreviations: IQR, interquartile range; SD, standard deviation.

^a^ Creole and Dutch (n = 1), Chinese and Creole (n = 1), Indonesian and Dutch (n = 2).

^b^ Physician diagnosis.

^c^ Positive patch test: remaining patients were never tested, unknown or tested negative.

^d^ Patient‐reported food allergy.

^e^ Patients who were employed, self‐employed, employed and self‐employed or employed and student or pupil at baseline.

^f^ Of the working patients: (self‐)employed patients who reported problems at work.

^g^ Pruritus and fatigue (n = 1), pruritus, fatigue and pain (n = 2), pruritus, fatigue and psychological problems (n = 2), pruritus, fatigue, pain and inconsistent course of illness (n = 2), pruritus, psychological problems and inconsistent course of illness (n = 1), pain and inconsistent course of illness (n = 1), pruritus, fatigue and other: eczema flare with stress (n = 1), psychological problems and other: visibility of the disease (n = 1), pain and other: eczema located on fingertips (n = 1), pruritus, fatigue and other: eye complaints (n = 1), pruritus, fatigue, pain and other: scaling/flaking skin (n = 1), pruritus, fatigue, receiving insufficient understanding from the working environment and other: tingling/burning skin sensation (n = 1).

^h^ Concentration problems (n = 1), planning of emollient use (n = 1), tight feeling of the skin and visibility of the disease (n = 1), the use of soap triggers eczema (n = 1).

^i^ Average number of days per month in the past 3 months.

^j^ In patients that reported days lost from usual activities.

^k^ n = 72.

^l^ n = 21.

Work Ability Index and QWLQ assessments were completed by 72 patients with a median follow‐up of 27.5 weeks (range, 0–100). At 48 weeks, data was available for 37 of the 72 patients. Due to the daily practice setting, seven visits occurred outside the aspired window, ranging from windows of 5 weeks in five patients, 7 weeks in one patient, to 8 weeks in one patient. No patients were lost to follow‐up.

### 3.1 Work ability

**FIGURE 1:**
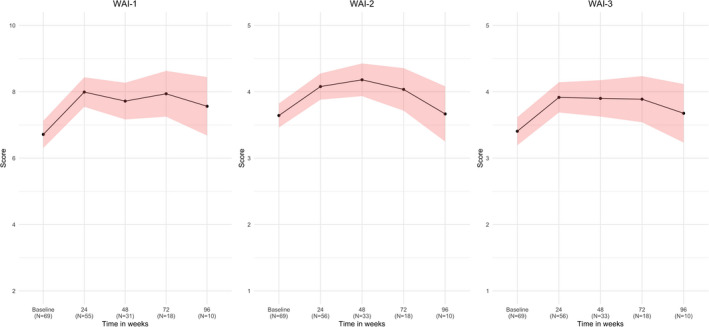
Work Ability Index (WAI) mean scores over time from baseline to 96 weeks of follow‐up. Results based on our linear mixed‐effects models. Higher scores indicate better patient‐reported general (WAI‐1), physical (WAI‐2), and mental (WAI‐3) work ability. The red area surrounding the black line represents the 95% confidence interval. To increase the legibility of this figure, data from the visits at 4 weeks of three patients were considered baseline data. Missing data: n = 6 at baseline; n = 3 for WAI‐1, n = 2 for WAI‐2 and WAI‐3 at 24 weeks; n = 6 for WAI‐1, n = 4 for WAI‐2 and WAI‐3 at 48 weeks; n = 1 at 72 weeks. As follow‐up duration varied between patients, the number of patients per visit decreases over time

Work Ability Index scores are shown in Figure 1. Improvement is observed from baseline, with a slight decrease as time progresses. At baseline, mean WAI‐1 indicating general work ability was 6.8 ± 2.0. The mean WAI‐2 indicating physical work ability was 3.7 ± 0.9. The mean WAI‐3 indicating mental/emotional work ability was 3.4 ± 0.9. Compared to baseline, the mean scores at 48 weeks significantly improved to 7.9 ± 1.3, 4.3 ± 0.7, and 3.9 ± 0.8 for WAI‐1, WAI‐2, and WAI‐3 respectively (*p* = 0.001, *p* = 0.005, *p* < 0.001).

### 3.2 Quality of working life

**FIGURE 2:**
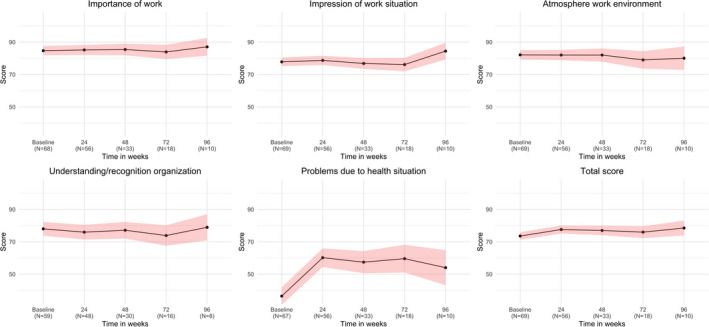
Quality of Working Life Questionnaire (QWLQ) mean (sub)scores over time from baseline to 96 weeks of follow‐up. Results based on our linear mixed‐effects models. Higher scores indicate better patient‐reported quality of working life. The red area surrounding the black line represents the 95% confidence interval. To increase the legibility of this figure, data from the visits at 4 weeks of three patients were considered baseline data. Missing data: n = 7, n = 6, n = 6, n = 16, n = 8, n = 6 for subscale 1 to total score, respectively at baseline; n=10 for subscale 4, n = 2 for the other scores at 24 weeks; n = 7 for subscale 4, n = 4 for the other scores at 48 weeks; n = 2 for subscale 4 at 72 weeks and 96 weeks. As follow‐up duration varied between patients, the number of patients per visit decreases over time.

Quality of Working Life Questionnaire scores are shown in Figure 2. The subscale “Problems due to health situation” was found to have the lowest mean of 37.4 ± 22.3, showing an increase followed by a slight decrease over time. The subscale with the highest baseline score was “Meaning of work” with a mean score of 85.2 ± 13.3, which remained stable over time. The subscale “Understanding and recognition in the organization” showed a decrease from a baseline score of 78.9 ± 14.9. Both the subscale “Perception of the work situation” and “Atmosphere in the working environment” showed a decrease in mean score from baseline (81.3 ± 12.9 and 82.3 ± 11.5, respectively), followed by an increase. The mean QWLQ total score was 74.0 ± 9.1 at baseline, 78.5 ± 9.8 at 24 weeks, 77.5 ± 9.6 at 48 weeks, 72.9 ± 13.1 at 72 weeks, and 76.4 ± 13.2 at 96 weeks. When comparing the means at baseline with 48 weeks, we only found significant improvement for total score and subscale “Problems due to the health situation” (4.1 points with *p* = 0.032 and 23.3 points with *p* < 0.001, respectively).

### 3.3 **Characteristics associated with change in QWLQ from baseline to 48** **weeks**

Table 2 shows the baseline characteristics significantly associated with change in score from baseline to 48 weeks (complete results shown in Table S1). We found that females reported more improvement of subscales “Meaning of work” (12.2 ± 4.5, *p* = 0.018) and “Atmosphere in the working environment” (12.0 ± 4.4, *p* = 0.021), and QWLQ total score (9.7 ± 4.0, *p* = 0.038) compared to males, whereas Asian patients had less improvement of the subscales “Perception of the work situation” (−12.8 ± 3.1, *p* < 0.001) and “Understanding and recognition in the organization” (−29.5 ± 9.6, *p* = 0.027), and the QWLQ total score (−13.2 ± 4.8, *p* = 0.022) compared to White patients. In addition, less improvement was observed for patients who experienced days lost from usual activities in subscales “Meaning of work” (−16.5 ± 6.5, *p* = 0.029) and “Atmosphere in the working environment” (−22.8 ± 6.1, *p* = 0.004). Patients with ISCED 2–4 and ISCED 5–6 had higher improvement of subscale “Atmosphere in the working environment” (36.3 ± 9.5, *p* = 0.003 and 29.1 ± 8.3, *p* = 0.006, respectively), compared to ISCED 0–1 patients. Patients who reported problems at work had higher improvement of subscale “Problems due to the health situation” (24.7 ± 9.3, *p* = 0.016) in comparison to patients who did not. Patients with allergic rhinoconjunctivitis had higher improvement of “Perception of the work situation” (9.3 ± 2.8, *p* = 0.005). Patients with atopic eye disease, contact dermatitis, and food allergy had lower improvement of respective subscales “Perception of the work situation” (−12.6 ± 4.2, *p* = 0.009), “Problems due to the health situation” (−33.5 ± 13.0, *p* = 0.020), and “Atmosphere in the working environment” (−17.2 ± 5.9, *p* = 0.016).

**TABLE 2:** Characteristics significantly associated with change in Quality of Working Life Questionnaire (QWLQ) (sub)scores from baseline to 48 weeks of follow‐up

Characteristics significantly associated with change in QWLQ (sub)scores from baseline to 48 weeks of follow‐up			
(Sub)scale	Characteristics	Estimated difference in score ± SE	*p*
Meaning of work	Female	12.2 ± 4.5	0.018
	Days lost from usual activities	−16.5 ± 6.5	0.029
Perception of the work situation	Asian	−12.8 ± 3.1	<0.001
	Allergic rhinoconjunctivitis	9.3 ± 2.8	0.005
	Atopic eye disease	−12.6 ± 4.2	0.009
Atmosphere in the working environment	ISCED 2–4	36.3 ± 9.5	0.003
	Days lost from usual activities	−22.8 ± 6.1	0.004
	ISCED 5–6	29.1 ± 8.3	0.006
	Food allergy	−17.2 ± 5.9	0.016
	Female	12.0 ± 4.4	0.021
	Allergic rhinoconjunctivitis	12.8 ± 5.8	0.052^a^
	Asthma	10.8 ± 5.0	0.056 ^a^
Understanding and recognition in the organization	Asian	−29.5 ± 9.6	0.027
Problems due to the health situation	Patient‐reported problems at work	24.7 ± 9.3	0.016
	Contact dermatitis	−33.5 ± 13.0	0.020
	ISCED 7–8	−41.7 ± 20.5	0.059 ^a^
Total score	Asian	−13.2 ± 4.8	0.022
	Female	9.7 ± 4.0	0.038
	Days lost from usual activities	−13.0 ± 6.0	0.060 ^a^

Notes

The reference standard was characteristic “not present” or “White” in case of “Asian”, “Male” in case of “Female”, “Unknown” in case of patch test/contact dermatitis, and “ISCED 0–1” in all ISCED variables.

Abbreviation: SE, standard error.

^a^ Borderline significant. Results are based on our multivariate models.

### 3.4 QWLQ convergent validity

Spearman correlations for the total QWLQ are shown in Table 3, with corresponding scatter plots in Figure S1A and S1B. For all PROMs, no correlations were found (*p*>0.05) and correlation coefficients did not exceed |0.40|. Only a borderline significant weak correlation was found for DLQI (*r* = −0.24, *p* = 0.058).

**TABLE 3:** Spearman correlation coefficients for the Quality of Working Life Questionnaire (QWLQ) total score in relation with comparable constructs at baseline

Convergent validity of the QWLQ total score: Spearman correlation coefficients		
Comparable construct	Spearman correlation coefficient (*r*)	*p*
EQ‐5D‐5L health state	0.17	0.186
POEM	0.00	1.000
DLQI	−0.24	0.058
PGA	0.04	0.764
NRS peak itch 0–10 past 24 h	0.05	0.674
NRS mean itch 0–10 past 7 days	−0.07	0.603
VAS peak pain 0–10 past 24 h	−0.08	0.513
VAS mean sleep loss 0–10 past 3 days	−0.13	0.303

Abbreviations: DLQI, Dermatology Life Quality Index; EQ‐5D‐5L, EuroQol‐5 dimensions‐5 level; NRS, Numerical Rating Scale; PGA, Patient Global Assessment; POEM, Patient‐Oriented Eczema Measure; QWLQ, Quality of Working Life Questionnaire; VAS, Visual Analogue Scale.

Table 4 shows Spearman correlations of QWLQ subscale 5 “Problems due to the health situation” (scatter plots: Figure S2A and S2B). We found a moderate positive correlation for EQ‐5D‐5L health state (*r* = 0.43, *p* < 0.001) and a strong negative correlation for DLQI (*r* = −0.65, *p* < 0.001). In addition, weak negative correlations were found for VAS peak pain and mean sleep loss (*r* = −0.26, *p* = 0.035 and *r* = −0.28, *p* = 0.023, respectively).

**TABLE 4:** Spearman correlation coefficients for the Quality of Working Life Questionnaire (QWLQ) subscale 5 ‘Problems due to the health situation’ in relation with comparable constructs at baseline

Convergent Validity of QWLQ subscale 5: Spearman correlation coefficients		
Comparable construct	Spearman correlation coefficient (*r*)	*p*‐Value
EQ‐5D‐5L health state	0.43	**<0.001**
POEM	−0.20	0.111
DLQI	−0.65	**<0.00001**
PGA	0.06	0.673
NRS peak itch 0–10 past 24 h	−0.02	0.873
NRS mean itch 0–10 past 7 days	−0.03	0.813
VAS peak pain 0–10 past 24 h	−0.26	**0.035**
VAS mean sleep loss 0–10 past 3 days	−0.28	**0.023**

Notes

Significant values are displayed in bold.

Abbreviations: DLQI, Dermatology Life Quality Index; EQ‐5D‐5L, EuroQol‐5 dimensions‐5 level; NRS, Numerical Rating Scale; PGA, Patient Global Assessment; POEM, Patient‐Oriented Eczema Measure; QWLQ, Quality of Working Life Questionnaire; VAS, Visual Analogue Scale.

### 3.5 Floor and ceiling effects

There were no patients in whom the lowest possible QWLQ total score (0) was observed. The highest possible QWLQ total score was found once in one patient (1/201 observations). A ceiling effect was observed only for subscale “Meaning of work” where in 41 out of 201 observations (20%) the highest score (100) was observed.

## 4 DISCUSSION

In this study we analyzed work‐related patient characteristics of 93 AD patients treated with dupilumab in daily practice. We primarily aimed to describe the longitudinal work ability and QWL of this population. Our patients reported a decreased work ability and QWL at baseline, mainly due to health‐related problems. Significant improvement of work ability and QWL was observed with treatment after 48 weeks. Furthermore, we assessed associations between patient characteristics and change in QWLQ and the convergent validity of the QWLQ.

The majority of working patients reported problems at work. In most cases, a combination of problems was reported, including pruritus, fatigue, pain, and psychological complaints. In earlier research, fatigue was found the main reason for work productivity loss in inflammatory bowel disease (IBD).^30^ In half of the employed IBD population, disease activity and disease burden was found to cause work productivity loss, driving indirect costs.^30^ It has been shown that the majority of moderate‐to‐severe AD patients miss at least 1 day of work per year.^31^ We found that more than half of our working patients reported days lost from usual activities (3.5 median days/month), indicating potential work productivity loss. Another study in AD patients showed a mean of 9.6–19 h/week work productivity loss.^32^

Regarding WAI, we found a decreased mean general work ability of 6.8 (0–10) and a mean physical and mental/emotional work ability of 3.7 and 3.4 (1–5) at baseline, respectively, with significant improvement at 48 weeks. In other studies, a mean general work ability of 5.1 was found in cancer survivors and of 5.4 in cancer patients at the time of diagnosis.^33^, ^34^ In contrast, a mean general work ability ranging 7.8–8.2 was found in nurses.^35^ In other chronic diseases, common prognostic factors for work disability were health complaints, limitation in daily physical activities caused by the disease, heavy manual work, and female sex.^36^

At baseline, we observed a mean QWLQ total score of 74.0, together with a mean score of 37.4 for subscale 5 “Problems due to the health situation”. In cancer survivors, a mean QWLQ total score of 75 and subscale 5 of 57 has been demonstrated, in contrast to a mean QWLQ total score of 79 and subscale 5 of 81 in employed people without cancer.^12^ In IBD patients, a mean QWLQ total score of 78 and subscale 5 of 54 was found.^37^ The results for the other subscales were similar between our AD, and cancer survivor and IBD populations.^12^, ^37^ The remarkably lower score for subscale 5 in our population shows that patients with AD experience a relatively high QWL burden regarding their health situation. The overall decrease in QWL is shown to be mainly driven by this subscale. We found significant and clinically meaningful improvement of the scores at 48 weeks.^13^ Greater improvement was observed in females compared to males.

Quality criteria have been defined for measurement properties of questionnaires, including convergent validity.^26^ A positive rating for construct validity is given if at least 75% of results correspond to a priori hypotheses.^38^ While our sample size was adequate (i.e. n = 50–99),^38^ we found no significant correlations between the QWLQ total score and the other PROMs. Thus, none of our hypotheses regarding moderate‐to‐strong correlations were confirmed. More suitable comparable constructs may be available (e.g., VAS overall QWL). Regardless, QWL seems not to be captured by broadly used validated PROM in AD and therefore the QWLQ could be considered of added value. We found a strong negative correlation between DLQI and QWLQ subscale 5, implicating that perceived health problems are accompanied by a decrease of quality of life.

Limitations of this study include several factors resulting from the daily practice setting. Non‐compliance and unintended dosing deviations are potential factors, as patients received their treatment at home. Bias may have been induced by the non‐blinded observational nature of the study. We did not focus on strict label use of dupilumab, and patients that used comedication or continued treatment in an alternative dosing schedule due to ineffectiveness or side‐effects were included in our analyses.

### 4.1 Implications for research and clinical practice

Further investigation of work ability and QWL using WAI and QWLQ in a larger population and comparing different treatments would be of interest. In the future, QWLQ could be used at a group level as effect measurement of interventions in research, as well as on individual patient level to monitor different aspects of QWL and to intervene with supportive care if appropriate. The latter strategy may facilitate to identify patients that benefit from tailored interventions. A need exists for development of programs that can support this demand. Furthermore, investigating the impact on work productivity specifically can contribute to determining the cost‐effectiveness of treatments.

### 4.2 Conclusion

In conclusion, the majority of AD patients starting with dupilumab, indicating moderate‐to‐severe disease, experience days lost from work and other usual activities, demonstrating potential work productivity loss. Most working patients report problems at work, often a combination of pruritus, fatigue, pain, and psychological complaints. Patients report a decreased work ability and experience a high burden regarding QWL, in particular due to health‐related problems. There seems to be significant improvement of work ability and QWL with dupilumab treatment over time.

## CONFLICT OF INTEREST

M. A. M.: consultancies for Sanofi and Pfizer; P. S.: consultancies in the past for Sanofi 111017 and AbbVie 041217 (unpaid), received a departmental independent research grant for TREAT NL registry from LeoPharma 2019, and Novartis in 2020 (and more companies agreed already to sponsor in order to have multisponsoring) for the TREAT NL registry, is involved in performing clinical trials with many pharmaceutical industries that manufacture drugs used for the treatment of, for example, psoriasis and atopic dermatitis, for which financial compensation is paid to the department/hospital, and is chief investigator of the systemic and phototherapy atopic eczema registry (TREAT NL) for adults and children, and one of the main investigators of the SECURE‐AD registry. All other authors declare that they have no conflicts of interest.

## Supporting information

Appendix S1

Appendix S2

Fig S1A

Fig S1B

Fig S2A

Fig S2B

Table S1

Supplementary Material
