# Supplementary material for: Work ability and quality of working life in atopic dermatitis patients treated with dupilumab
Source: J Dermatol. 2021 May 19;48(9):1305–14. doi: 10.1111/1346-8138.15939 (PMC8453967; doi:10.1111/1346-8138.15939)
Supplement: Supplementary file 1 — Appendix S1 [file JDE-48-1305-s001.docx]

Appendix S1. Work Ability Index (WAI): first three questions (WAI-1, WAI-2, WAI-3):

1. Assume that your work ability at its best has a value of 10 points. How many points would you give your current work ability? (0 means that you currently cannot work at all)

0 1 2 3 4 5 6 7 8 9 10

↑ ↑

No work ability at all Best work ability ever

1. How do you rate your current work ability with respect to the physical demands of your work?
2. Very poor
3. Rather poor
4. Moderate
5. Rather good
6. Very good
7. How do you rate your current work ability with respect to the mental demands of your work?
8. Very poor
9. Rather poor
10. Moderate
11. Rather good
12. Very good
