# Supplementary material for: Work ability and quality of working life in atopic dermatitis patients treated with dupilumab
Source: J Dermatol. 2021 May 19;48(9):1305–14. doi: 10.1111/1346-8138.15939 (PMC8453967; doi:10.1111/1346-8138.15939)
Supplement: Supplementary file 2 — Appendix S2 [file JDE-48-1305-s008.docx]

Appendix S2. Quality of Working Life Questionnaire (QWLQ)

**INSTRUCTIONS for employees**

This questionnaire is about your experience and perception of your work during the **past four weeks**. In order to answer the questionnaire it is important, therefore, that you worked at some time during the past four weeks.

The questions in the questionnaire can be answered with ‘agree’ or ‘disagree’. There are no right or wrong answers. If you are unsure how to answer a question, give the best answer you can. Do not skip any questions. All your answers to the questions will be treated in confidence, and the results will be processed anonymously.

The questions are about your current work situation. You should therefore try to give answers about the **past four weeks**. If you have more than one job, give answers for the job in which you work the most hours.

**INSTRUCTIONS for self-employed persons**

This questionnaire is about your experience and perception of your work during the **past four weeks**. In order to answer the questionnaire it is important, therefore, that you worked at some time during the past four weeks.

The questions in the questionnaire can be answered with ‘agree’ or ‘disagree’. There are no right or wrong answers. If you are unsure how to answer a question, give the best answer you can. Do not skip any questions. All your answers to the questions will be treated in confidence, and the results will be processed anonymously.

The questions are about your current work situation. You should therefore try to give answers about the **past four weeks**. If you have more than one job, give answers for the job in which you work the most hours.

It may be that a question does not apply to you, for example because you do not have an immediate superior. In that case you can use the answer category ‘not applicable’ (N/A).

**The following questions are about the meaning of work.**

Please indicate the extent to which you agree or disagree with the following statements in relation to the past four weeks. Tick one choice for each statement.

|  | **Disagree completely** | **Disagree** | **Disagree slightly** | **Agree slightly** | **Agree** | **Agree completely** |
| --- | --- | --- | --- | --- | --- | --- |
| 1. Working gives me structure in my life |  |  |  |  |  |  |
| 1. I think it is good to work |  |  |  |  |  |  |
| 1. I consider that my work gives me a goal in life |  |  |  |  |  |  |
| 1. I consider my work important |  |  |  |  |  |  |

**The following questions are about your perception of your work situation.**

Please indicate the extent to which you agree or disagree with the following statements in relation to the past four weeks. Tick one choice for each statement.

|  | **Disagree completely** | **Disagree** | **Disagree slightly** | **Agree slightly** | **Agree** | **Agree completely** |
| --- | --- | --- | --- | --- | --- | --- |
| 1. I do my work well |  |  |  |  |  |  |
| 1. I am self-confident in my work |  |  |  |  |  |  |
| 1. I am suited to my work |  |  |  |  |  |  |
| 1. I have control over the work I do |  |  |  |  |  |  |
| 1. I feel powerless in my work |  |  |  |  |  |  |

**The following questions are about the atmosphere in your working environment.**

Please indicate the extent to which you agree or disagree with the following statements in relation to the past four weeks. Tick one choice for each statement.

*If the statement has not applied in your work situation as a self-employed person, give the answer ‘N/A’ (not applicable).*

|  | **Disagree completely** | **Disagree** | **Disagree slightly** | **Agree slightly** | **Agree** | **Agree completely** | **NA** |
| --- | --- | --- | --- | --- | --- | --- | --- |
| 1. I feel there is a positive atmosphere in my working environment |  |  |  |  |  |  |  |
| 1. I have the feeling I am taken seriously by people in my working environment |  |  |  |  |  |  |  |
| 1. I am content with my work |  |  |  |  |  |  |  |
| 1. I have good relations with my colleagues |  |  |  |  |  |  |  |
| 1. I feel valuable to my colleagues |  |  |  |  |  |  |  |

**The following questions are about understanding and recognition in your organization.**

Please indicate the extent to which you agree or disagree with the following statements in relation to the past four weeks. Tick one choice for each statement.

*If the statement has not applied in your work situation as a self-employed person, give the answer ‘N/A’ (not applicable).*

|  | **Disagree completely** | **Disagree** | **Disagree slightly** | **Agree slightly** | **Agree** | **Agree completely** | **NA** |
| --- | --- | --- | --- | --- | --- | --- | --- |
| 1. My immediate superior understands my health situation and possible health problems |  |  |  |  |  |  |  |
| 1. I have good relations with my immediate superior |  |  |  |  |  |  |  |
| 1. I consider that employees with health problems are treated well in my organization |  |  |  |  |  |  |  |
| 1. I am content with the fringe benefits provided by my employer |  |  |  |  |  |  |  |
| 1. I am content with my current income |  |  |  |  |  |  |  |

**The following questions are about problems due to your health situation.**

Please indicate the extent to which you agree or disagree with the following statements in relation to the past four weeks. Tick one choice for each statement.

|  | **Disagree completely** | **Disagree** | **Disagree slightly** | **Agree slightly** | **Agree** | **Agree completely** |
| --- | --- | --- | --- | --- | --- | --- |
| 1. Because of my health situation I have problems in my work with fatigue and/or lack of energy |  |  |  |  |  |  |
| 1. I am limited in my work by my health situation |  |  |  |  |  |  |
| 1. Because of my health situation I have little trust in my own body |  |  |  |  |  |  |
| 1. Because of my health situation I feel uncertain about the future |  |  |  |  |  |  |
