# Supplementary figures and images for: Work ability and quality of working life in atopic dermatitis patients treated with dupilumab
Source: J Dermatol. 2021 May 19;48(9):1305–14. doi: 10.1111/1346-8138.15939 (PMC8453967; doi:10.1111/1346-8138.15939)

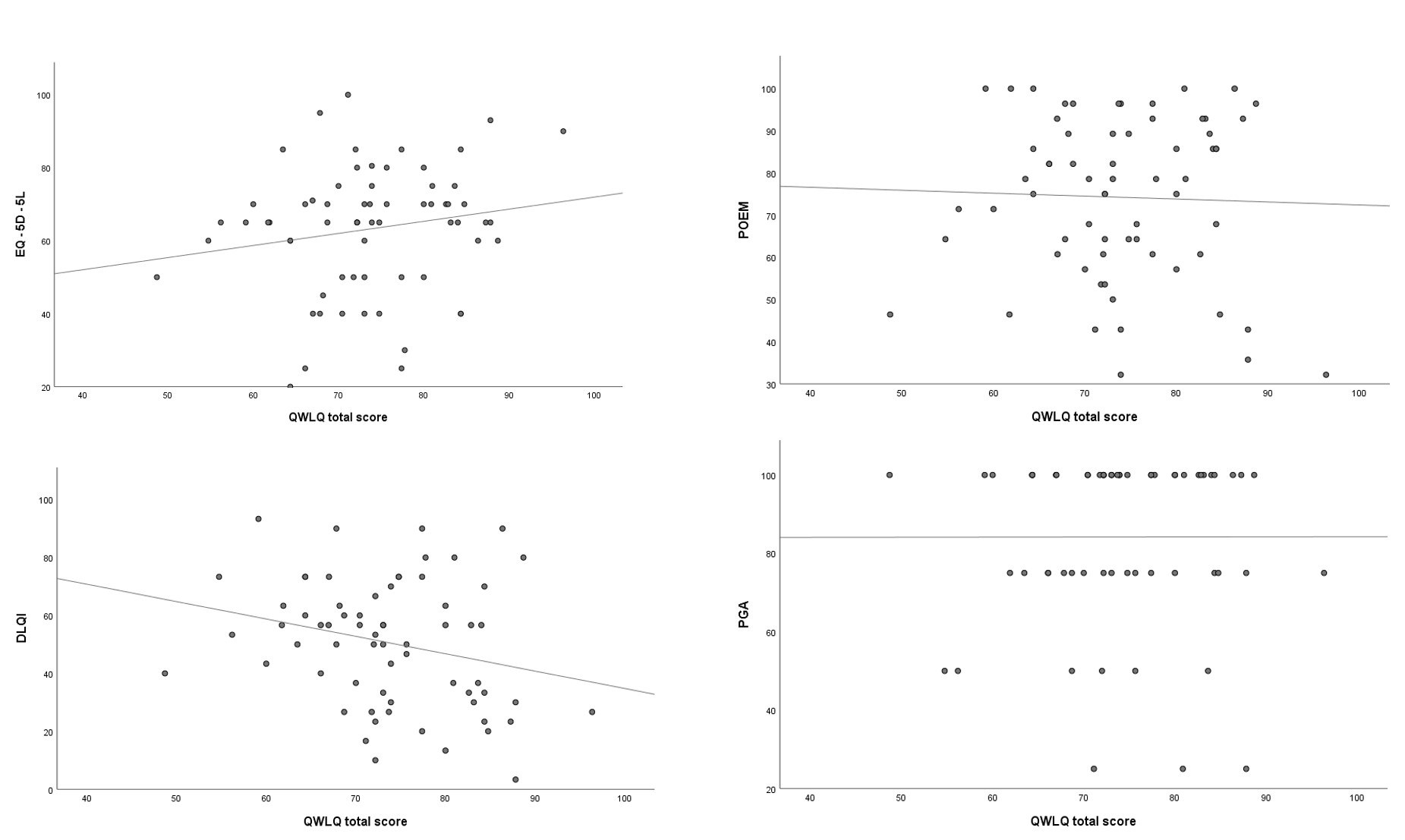

Supplement: Supplementary file 3 — Fig S1A [file JDE-48-1305-s007.jpg]

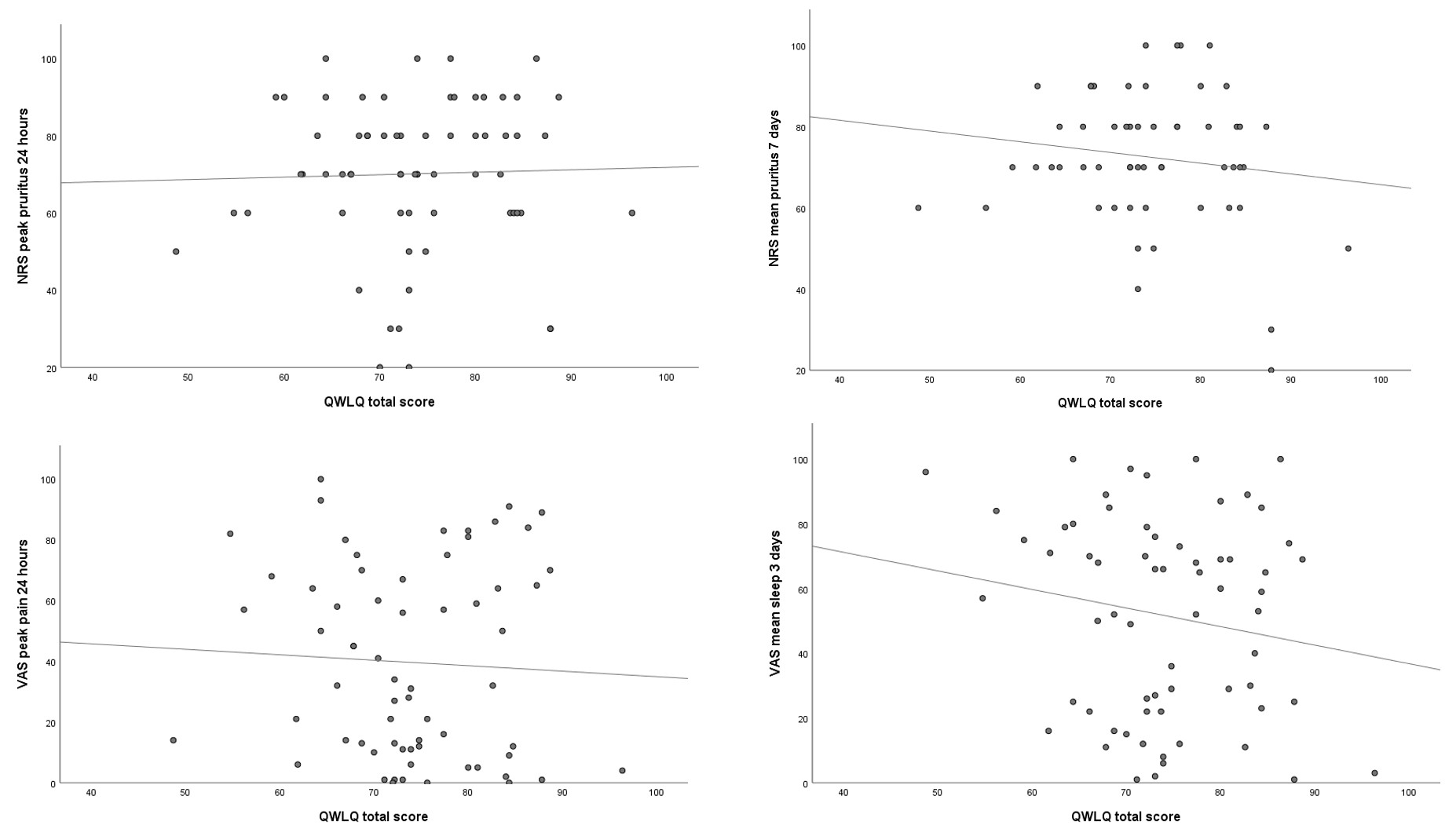

Supplement: Supplementary file 4 — Fig S1B [file JDE-48-1305-s004.jpg]

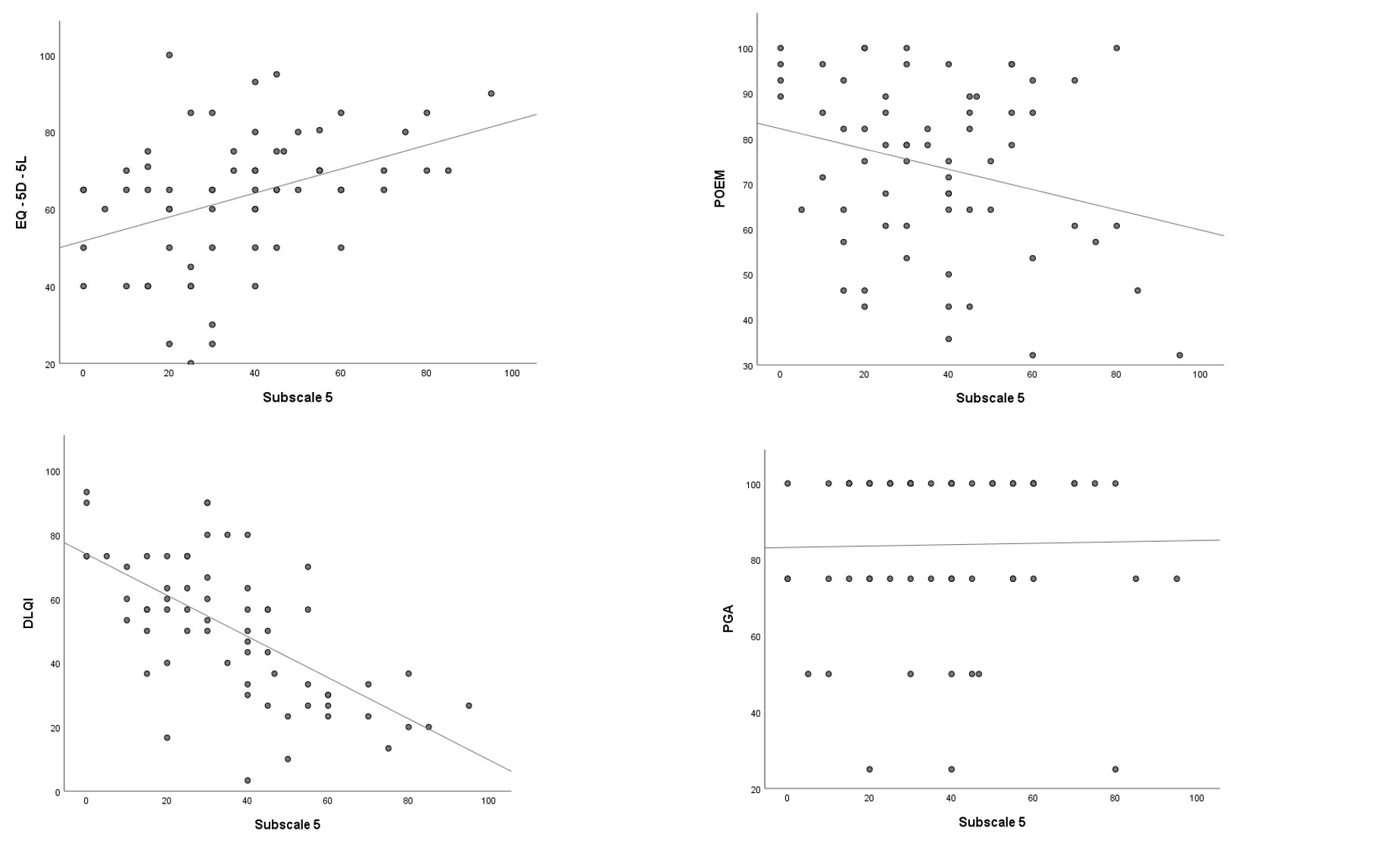

Supplement: Supplementary file 5 — Fig S2A [file JDE-48-1305-s002.jpg]

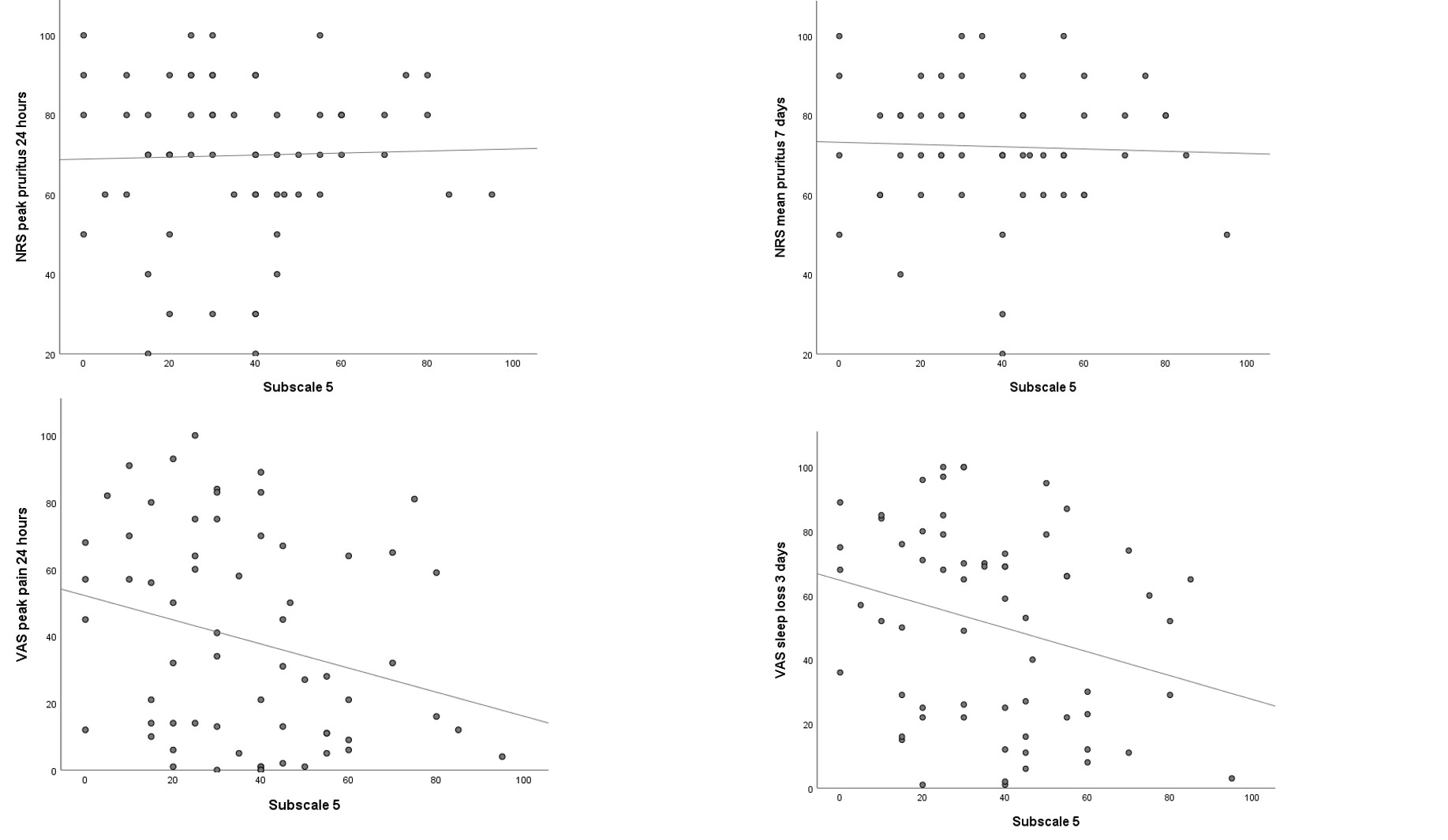

Supplement: Supplementary file 6 — Fig S2B [file JDE-48-1305-s003.jpg]
