# Supplementary material for: Work ability and quality of working life in atopic dermatitis patients treated with dupilumab
Source: J Dermatol. 2021 May 19;48(9):1305–14. doi: 10.1111/1346-8138.15939 (PMC8453967; doi:10.1111/1346-8138.15939)
Supplement: Supplementary file 7 — Table S1 [file JDE-48-1305-s005.docx]

Table S1. Characteristics in the multivariate models for change in Quality of Working Life Questionnaire (QWLQ) (sub)scores from baseline to 48 weeks

| Characteristics in the multivariate models for change in QWLQ (sub)scores from baseline to 48 weeks | | |
| --- | --- | --- |
| Meaning of work | Estimate (SE) | p-value |
| Age | 0.2 (0.2) | 0.273 |
| Male gender | - | **-** |
| Female gender | 12.2 (4.5) | **0.018** |
| White ethnicity | - | - |
| Black ethnicity | -9.5 (12.0) | 0.445 |
| Asian ethnicity | -9.2 (5.4) | 0.111 |
| Other ethnicity | -9.1 (9.6) | 0.362 |
| Allergic rhinoconjunctivitis | 6.4 (4.5) | 0.178 |
| Atopic eye disease | -13.5 (7.2) | 0.089 |
| Unknown patch test | - | - |
| Positive patch test | 5.3 (7.3) | 0.489 |
| Negative patch test | 0.1 (12.4) | 0.992 |
| ISCED 0-1 | - | - |
| ISCED 2-4 | 13.7 (8.5) | 0.129 |
| ISCED 5-6 | 3.7 (8.4) | 0.670 |
| ISCED 7-8 | -1.7 (10.0) | 0.871 |
| Days lost from usual activities | -16.5 (6.5) | **0.029** |
| Patient-reported problems at work | 9.2 (6.9) | 0.220 |
| Perception of the work situation |  |  |
| Male gender | - | - |
| Female gender | 3.3 (3.1) | 0.307 |
| White ethnicity | - | - |
| Black ethnicity | -11.7 (9.0) | 0.216 |
| Asian ethnicity | -12.8 (3.1) | **<0.001** |
| Other ethnicity | 4.1 (6.6) | 0.546 |
| Allergic rhinoconjunctivitis | 9.3 (2.8) | **0.005** |
| Atopic eye disease | -12.6 (4.2) | **0.009** |
| Unknown patch test | - | - |
| Positive patch test | -6.3 (4.5) | 0.184 |
| Negative patch test | -0.4 (7.1) | 0.955 |
| Patient-reported food allergy | -2.9 (3.4) | 0.407 |
| Days lost from usual activities | -3.9 (4.6) | 0.412 |
| Patient-reported problems at work | 6.3 (5.1) | 0.250 |
| Atmosphere in the working environment |  |  |
| Male gender | - | **-** |
| Female gender | 12.0 (4.4) | **0.021** |
| Age | 0.1 (0.2) | 0.509 |
| White ethnicity | - | - |
| Black ethnicity | 14.6 (13.0) | 0.290 |
| Asian ethnicity | -6.2 (5.4) | 0.274 |
| Other ethnicity | 3.8 (9.6) | 0.697 |
| Asthma | 10.8 (5.0) | 0.056* |
| Allergic rhinoconjunctivitis | 12.8 (5.8) | 0.052* |
| Unknown patch test | - | - |
| Positive patch test | 3.2 (6.7) | 0.641 |
| Negative patch test | -4.2 (11.0) | 0.717 |
| Patient-reported food allergy | -17.2 (5.9) | **0.016** |
| ISCED 0-1 | - | **-** |
| ISCED 2-4 | 36.3 (9.5) | **0.003** |
| ISCED 5-6 | 29.1 (8.3) | **0.006** |
| ISCED 7-8 | 19.5 (10.5) | 0.092 |
| Occupation employed | - | - |
| Occupation self-employed | -9.2 (7.8) | 0.263 |
| Occupation other | -11.6 (8.2) | 0.186 |
| Days lost from usual activities | -22.8 (6.1) | **0.004** |
| Patient-reported problems at work | 1.6 (7.0) | 0.827 |
| Understanding and recognition in the organization |  |  |
| Male gender | - | - |
| Female gender | 9.2 (8.7) | 0.334 |
| Age | -0.4 (0.3) | 0.298 |
| White ethnicity | - | - |
| Black ethnicity | 1.2 (26.1) | 0.967 |
| Asian ethnicity | -29.5 (9.6) | **0.027** |
| Other ethnicity | -28.3 (39.1) | 0.568 |
| Asthma | -11.8 (13.4) | 0.424 |
| Allergic rhinoconjunctivitis | 6.0 (9.5) | 0.550 |
| Atopic eye disease | -13.6 (23.3) | 0.605 |
| Unknown patch test | - | - |
| Positive patch test | -26.2 (16.6) | 0.182 |
| Negative patch test | -5.3 (56.9) | 0.939 |
| Patient-reported food allergy | 8.4 (11.5) | 0.497 |
| ISCED 0-1 | - | - |
| ISCED 2-4 | 4.7 (20.7) | 0.831 |
| ISCED 5-6 | -2.6 (18.6) | 0.896 |
| ISCED 7-8 | 8.2 (25.1) | 0.766 |
| Occupation employed | - | - |
| Occupation other | -10.7 (28.0) | 0.733 |
| Days lost from usual activities | -12.3 (12.8) | 0.400 |
| Patient-reported problems at work | 13.3 (15.9) | 0.461 |
| Problems due to the health situation |  |  |
| Male gender | - | - |
| Female gender | 12.7 (8.9) | 0.168 |
| Atopic eye disease | -13.3 (17.7) | 0.467 |
| Unknown patch test | - | **-** |
| Positive patch test | -33.5 (13.0) | **0.020** |
| Negative patch test | -26.2 (16.2) | 0.124 |
| ISCED 0-1 | - | - |
| ISCED 2-4 | -16.6 (18.5) | 0.384 |
| ISCED 5-6 | -32.7 (19.2) | 0.108 |
| ISCED 7-8 | -41.7 (20.5) | 0.059* |
| Occupation employed | - | - |
| Occupation self-employed | -28.8 (15.3) | 0.078 |
| Occupation other | 3.1 (24.1) | 0.901 |
| Patient-reported problems at work | 24.7 (9.3) | **0.016** |
| Total score |  |  |
| Male gender | - | **-** |
| Female gender | 9.7 (4.0) | **0.038** |
| Age | -0.1 (0.2) | 0.775 |
| White ethnicity | - | - |
| Black ethnicity | 0.2 (11.9) | 0.980 |
| Asian ethnicity | -13.2 (4.8) | **0.022** |
| Other ethnicity | -0.8 (8.3) | 0.929 |
| Asthma | 4.3 (4.6) | 0.380 |
| Allergic rhinoconjunctivitis | 5.3 (5.2) | 0.336 |
| Atopic eye disease | -11.4 (8.0) | 0.189 |
| Unknown patch test | - | - |
| Positive patch test | -8.3 (6.6) | 0.239 |
| Negative patch test | -12.4 (10.3) | 0.261 |
| Patient-reported food allergy | -3.4 (5.3) | 0.537 |
| ISCED 0-1 | - | - |
| ISCED 2-4 | 9.2 (9.0) | 0.332 |
| ISCED 5-6 | 2.1 (8.4) | 0.805 |
| ISCED 7-8 | 2.7 (9.7) | 0.783 |
| Occupation employed | - | - |
| Occupation self-employed | -6.2 (7.5) | 0.431 |
| Occupation other | -7.6 (9.9) | 0.464 |
| Days lost from usual activities | -13.0 (6.0) | 0.060* |
| Patient-reported problems at work | 10.7 (6.6) | 0.143 |

The reference standard was characteristic ‘not present’ or ‘White’ in case of ethnicity, ‘Male’ in case of gender, ’Unknown’ in case of patch test/contact dermatitis, ‘Employed’ in case of occupation and ‘ISCED 0-1’ in all ISCED variables. *, borderline significant. Significant values are displayed in bold. Results are based on our multivariate models for each (sub)score. The variables that were included in the models: Age, Gender, Ethnicity (White, Black, Asian, Other), Patch test result (Positive, Negative, Unknown), Food allergy, Allergic rhinoconjunctivitis, Asthma, Atopic eye disease, ISCED classification (0-1, 2-4, 5-6, 7-8), Occupation (Employed, Self-employed, Other), Patient-reported problems at work, Days lost from usual activities.
