# Supplementary material for: Work ability and quality of working life in atopic dermatitis patients treated with dupilumab
Source: J Dermatol. 2021 May 19;48(9):1305–14. doi: 10.1111/1346-8138.15939 (PMC8453967; doi:10.1111/1346-8138.15939)
Supplement: Supplementary file 8 — Supplementary Material [file JDE-48-1305-s006.docx]

Supplementary figure legends

Figure S1A. Scatter plot with fit line for percentage QWLQ total score in relation to percentage EQ-5D-5L health state, POEM, DLQI and PGA at baseline

Figure S1B. Scatter plot with fit line for percentage QWLQ total score in relation to percentage NRS peak pruritus 24 hours, NRS mean pruritus 7 days, VAS peak pain 24 hours and VAS mean sleep 3 days at baseline

Figure S2A. Scatter plot with fit line for percentage QWLQ subscale 5 in relation to percentage EQ-5D-5L health state, POEM, DLQI and PGA at baseline

Figure S2B. Scatter plot with fit line for percentage QWLQ subscale 5 in relation to percentage NRS peak pruritus 24 hours, NRS mean pruritus 7 days, VAS peak pain 24 hours and VAS mean sleep 3 days at baseline

The comparable construct scores were converted into a percentage with 100% being the maximum score of the construct.
